# Supplementary figures and images for: Hemagglutinin Antibodies in the Polish Population during the 2019/2020 Epidemic Season
Source: Viruses. 2023 Mar 16;15(3):760. doi: 10.3390/v15030760 (PMC10052160; doi:10.3390/v15030760)

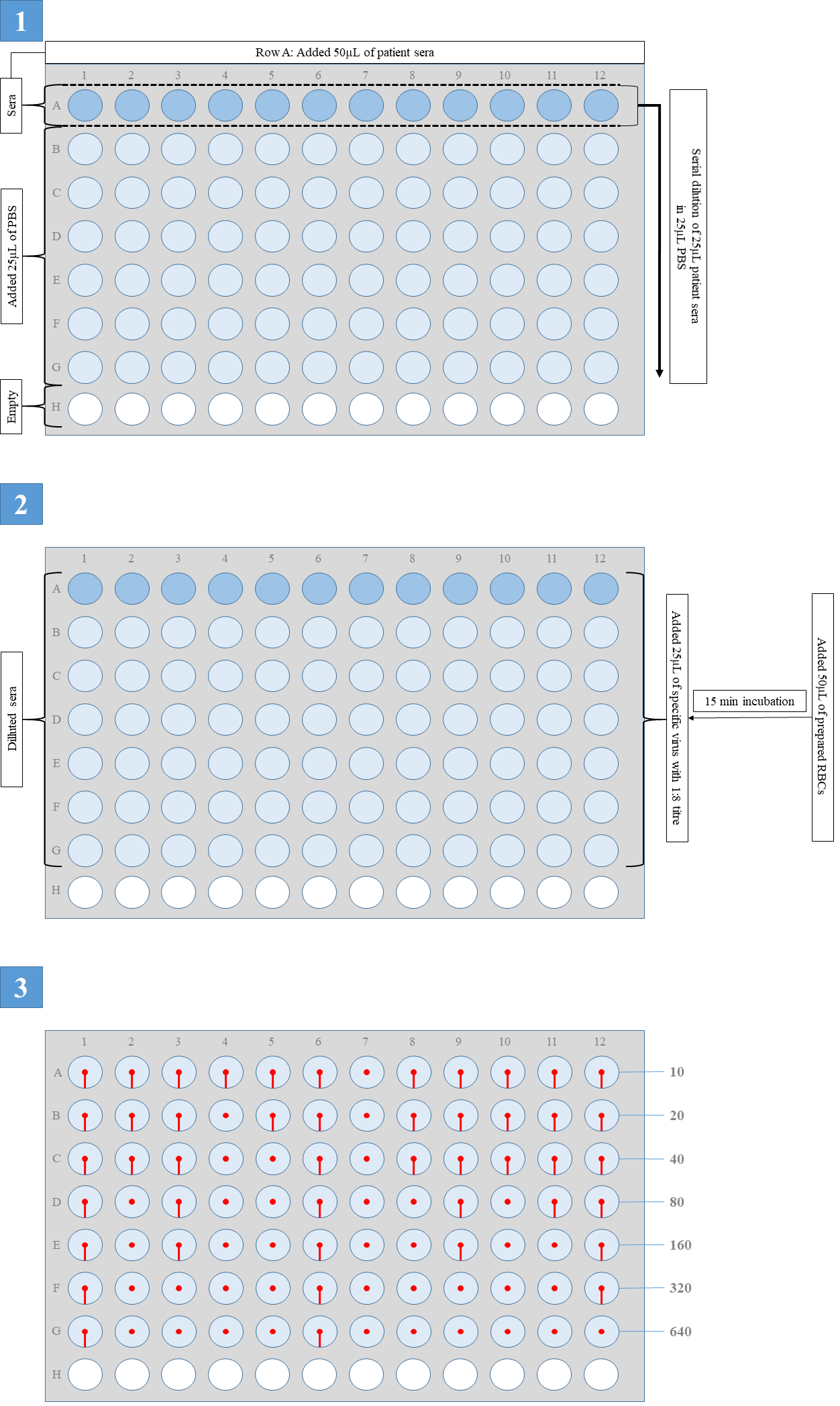

Supplement: Supplementary file 1 [file viruses-15-00760-s001.zip › viruses-2218590-supplementary.png]
